# Are we compulsively chasing rainbows?

**DOI:** 10.1038/s41386-022-01419-w

**Published:** 2022-08-18

**Authors:** Olivier George, Serge H. Ahmed, Nicholas W. Gilpin

**Affiliations:** 1 Department of Psychiatry, University of California, San Diego, CA USA; 2 University of Bordeaux, CNRS, INCIA, UMR 5287, EPHE, F-33000 Bordeaux, France; 3 Department of Physiology, Louisiana State University Health Sciences Center, New Orleans, LA USA; 4 Neuroscience Center of Excellence, Louisiana State University Health Sciences Center, New Orleans, LA USA; 5 Alcohol & Drug Abuse Center of Excellence, Louisiana State University Health Sciences Center, New Orleans, LA USA; 6 Southeast Louisiana VA Healthcare System (SLVHCS), New Orleans, LA USA

**Keywords:** Addiction, Reward

## Compulsive substance use in addiction

Addiction is commonly defined as a chronic, relapsing disorder characterized by taking drugs in excess, *compulsive* drug seeking, and continued use despite harmful consequences (NIDA, SAMSHA) despite the fact that the word “compulsive” is not explicitly stated in the DSM- or ICD-based diagnostic classification of substance use disorders (SUD), and that the role of compulsivity in addiction remains highly debated [1]. Similarly, in the preclinical addiction field, the idea that the only way to identify an individual with addiction-like behaviors is to measure compulsive drug use/drug seeking is pervasive and often treated as the only approach for studying neuropharmacological mechanisms relevant to addiction. Recently, the addiction neuroscience field has moved from recognizing that “compulsive drug seeking/use” and “continued seeking/use despite negative consequences” are two distinct aspects of addiction to defining the former nearly exclusively by the latter [2–7]. In our opinion, this informal but pervasive re-definition of compulsion has sacrificed construct validity for operationalization, and a direct consequence of this re-definition is that continued drug use (or seeking) despite punishment (e.g., painful footshock or bitter tastants) is widely considered *the* behavioral hallmark for compulsive-like drug-seeking behavior in preclinical models. We believe that over-reliance of addiction neuroscientists (including ourselves) on this measure hinges more on experimental convenience (i.e., testability) than on construct validity, and we also believe that over-reliance on this approach may be detrimental to the field. Here, we highlight several issues associated with the definition of compulsivity in addiction, problems associated with the regard for compulsivity as the defining feature of addiction, and the existence of conceptual and methodological limitations in preclinical studies. We also suggest ways for the field to move forward from the current position. Note that ‘drug’ in this commentary is a generic term that refers to alcohol, nicotine, and tobacco products, and illicit drugs.

## Issue #1: The definition of compulsive behavior does not require adverse consequences

Compulsion is a transdiagnostic psychiatric symptom that has been difficult to define [1, 8]. In our opinion, the most useful approach is to define compulsive behavior (not compulsion per se) as “repetitive acts that are characterized by the feeling that one ‘has to’ perform them while one is aware that these acts are not in line with one’s overall goal” [8]. This definition emphasizes a subjective experience in which an individual may feel compelled to repeat behaviors they do not want to repeat or even actively resist. In other words, compulsive behavior implies the presence of a motivational conflict that can be reported, but that is difficult to measure behaviorally in humans and it is unclear if this is even possible in animals [8]. Moreover, few if any definitions of compulsion/compulsive behavior explicitly mention the necessary presence of continued behavior despite associated adverse consequences. Indeed, many individuals with prototypical compulsive behaviors (e.g., Tourette syndrome, trichotillomania, and obsessive-compulsive disorders) can temporarily maintain some level of control over their compulsive behavior in situations that would lead to adverse consequences [9]. This demonstrates that magnitude of behavioral suppression in the presence of adverse consequences does not indicate the presence or absence of “compulsion”.

### Way forward

In all studies that measure X as a model of Y, one should avoid overinterpretation of findings as they relate to Y. Therefore, in preclinical studies that measure drug use despite adverse consequences, we recommend avoiding over-interpretation of findings as it relates to *compulsivity* and *addiction*. Instead, these results should be described as an indication of *persistent responding despite adverse consequences*. More effort should be made to better capture the motivational conflict at the heart of the concept of compulsive behavior. One way to achieve this goal is to obtain evidence that animals that use drugs despite adverse consequences are indeed conflicted (*e.g*., they are hesitant in responding for the drug, leading to increased latency to respond or increased aborted responses). Such conflicts are more likely to emerge in presence of increasing drug costs and/or in presence of competing non-drug alternatives. This general recommendation should also apply to any behavioral model in which researchers claim that the observed drug-reinforced responding reflects compulsivity, including the escalation model [10, 11].

## Issue #2: Drug use despite adverse consequences in animals with limited intoxication history is not equivalent to compulsive drug use in animals with chronic intoxication history

Compulsive drug use can contribute to drug use despite adverse consequences, but drug use in the face of adverse consequences is not synonymous with compulsive drug use and the two are dissociable over the course of addiction. Compulsive drug use occurs after chronic drug use and is often associated with mild to severe SUD, whereas continued drug use despite negative consequences (*e.g*., innapropriate behavior, belligerence, mood lability, impaired judgment) can be observed in individuals with limited drug intoxication history and is frequently observed in the absence of an SUD diagnosis. While one may argue that the neurobiological mechanisms underlying so-called compulsive-like responding in an individual with limited prior intoxication are relevant for addiction, there is abundant evidence that compulsive-like drug responding with limited drug intoxication may not be driven by the same mechanisms [12] and may not be responsive to the same treatments as compulsive-like drug responding with a history of chronic drug intoxication [13–16]. In addition, the neurobiological substrates underlying action-outcome associations are likely to differ according to (1) the extent of prior drug exposure and (2) whether or not acute intoxication is associated with negative consequences (which may be immediate or delayed).

### Way forward

It is critical for animal models to include chronic drug exposure histories that repeatedly achieve intoxication levels and blood-drug levels that are clinically relevant. It is also important that animal models compare adverse consequences of drug use that are immediate or delayed (and absent in controls).

## Issue #3: Persistent drug use despite adverse consequences can be explained by many factors other than compulsion

Drug use despite adverse consequence such as a punishment (or aversive signal) has important limitations and may be explained by other factors. *First*, animals that use drugs despite physical punishment may be less responsive to punishment because they are (1) less sensitive to nociceptive effects of physical punishment (i.e., footshock) [17], (2) more sensitive to footshock-induced antinociception [18], or (3) more resistant to the behavior-suppressing effects of punishment after chronic exposure to the punishing stimulus (or other stimuli) (e.g., rats acquire learned resistance) to footshock punishment [19]. *Second*, some individuals may use drugs despite physical punishment because they are more sensitive to the acute hypoalgesic or anti-hyperalgesic effect of the drug. *Third*, animals that consume drugs orally despite the presence of a typically aversive bitter tastant (e.g., quinine) may have less sensitivity to the bitter tastant either at baseline or as a result of chronic oral drug consumption. *Finally*, some individuals may use drugs despite punishment due to a deficit in learning the instrumental punishment contingency [20]. Unfortunately, the vast majority of preclinical studies do not control for these factors. For instance, in the last two years, only ~46% of studies using an adverse consequence tested if so-called resistant and vulnerable animals differed in innate sensitivity to the adverse consequence, none of the studies tested whether animals differed in sensitivity to the antinociceptive effect of the drug/shock, and none of the studies tested whether animals differed in their ability to learn the contingency (Supplementary Note 1). Even in an ideal scenario in which all above alternative explanations for drug use despite adverse consequences are ruled out, there is still the possibility that some individuals use a drug despite punishment because of a choice rather than a compulsion. In short, it is difficult, if not impossible, to ascertain that a behavior in an animal is “compulsive” or even “compulsive-like”.

### Way forward

In studies that measure drug use in the presence of adverse consequences, we recommend ruling out alternative explanations that include: delay discounting, baseline nociception, sensitivity to drug anti-nociceptive effects, learned resistance to punishment, tastant aversion, and instrumental contingency learning. We also recommend trying to generalize findings through the use of alternative measures (e.g., continued use despite a steep increase in cost, continued use despite an alternative choice). In the absence of these measures, we recommend avoiding use of the term “compulsive-like” to describe behavior in animals.

## Issue #4: Compulsivity is but one component of addiction

Focusing only on compulsive-like behavioral measures to identify individuals with addiction-like behaviors in preclinical studies ignores the heterogeneity of people with SUDs [21–23]. Even if one postulates that all humans with SUD exhibit compulsive drug use (which we know is not true), the psychological and biological mechanisms driving compulsivity in those individuals is likely to differ based on differences in peak intoxication, blood-drug levels, length of use, degree of tolerance to specific drug effects, intensity of withdrawal, presence of comorbid disorders, pre-existing conditions, and/or the relative importance of different drug reinforcement processes [24]. For instance, treatment efficacy may differ in patients seeking drug reward versus relief from negative states or symptoms [25, 26], and effective treatment may differ in patients with or without physical and emotional withdrawal symptoms resulting from prior drug use [23].

### Way forward

In studies that aim to identify the biological basis of compulsive-like drug use, we recommend using multidimensional phenotypic assessment in animals aimed at capturing the heterogeneity of addiction-like behaviors (e.g., physical withdrawal, affective-like behaviors, incentive salience, habit, hedonic allostasis, etc.) and identifying individual differences in mechanisms underlying different phenotypes, including but not limited to drug use despite adverse consequences.

## Issue #5: Equating compulsivity with addiction hinders treatment and harm reduction

Historically, the definition of addiction as a compulsion was thought to be helpful in reducing stigma and encouraging compassion and treatment of people with SUD. This approach has mostly failed. Only ~10% of people with SUD receive treatment, and stigmatization has not abated [27]. People recognize that they are using drugs excessively, but they are less likely to regard their drug use as “compulsive”. By equating compulsivity with addiction, scientists increase the likelihood that (1) individuals using drugs in excess but not “compulsively” will not seek treatment, and (2) health care providers will focus treatment efforts on individuals with compulsive drug use, which may miss a large group of individuals that would benefit from treatment. Treatment strategies that reduce drug consumption and promote harm reduction, independent of whether or not patients exhibit compulsive patterns of drug use, is more likely to positively impact human health than treatment strategies that focus on reducing “compulsive drug use”.

### Way forward

Addiction is a spectrum disorder that does not require the presence of compulsive drug use to be a significant health problem. We should broaden the base of treatment and increase efforts aimed at helping individuals to reduce harmful consumption whether or not those individuals exhibit compulsive drug use [28]. Preclinical studies should model different stages of addiction, including psychological and biological mechanisms underlying chronic heavy drug use with or without compulsive-like behavior.

## Conclusions

It is critical to recognize that the concept of compulsivity in addiction remains highly debated in both the clinical and preclinical fields and that, while useful, preclinical models of drug use despite adverse consequences have major conceptual and methodological limitations. While we encourage their use in a multidimensional phenotypic assessment approach aimed at capturing the heterogeneity of addiction-like behaviors, we strongly discourage researchers from equating compulsivity with addiction—they are not synonymous. Addiction is a heterogeneous spectrum disorder that cannot be reduced to compulsivity, and the idea that we can find a silver bullet for the treatment of addiction by focusing on compulsive-like behaviors is tantamount to chasing rainbows. To accelerate the development of new treatments for addiction, this heterogeneity should be acknowledged, embraced, investigated, and leveraged using a multidimensional approach that incorporates clinically relevant patterns of chronic drug exposure, routes of administration, and blood-drug levels. Finally, we should increase our efforts aimed at identifying behavioral and pharmacological approaches that reduce harmful drug consumption and drug choices whether or not such consumption or choice is associated with compulsive-like behavior.

## Supplementary information

Supplementary Note 1
