# Supplementary material for: Are we compulsively chasing rainbows?
Source: Neuropsychopharmacology. 2022 Aug 18;47(12):2013–5. doi: 10.1038/s41386-022-01419-w (PMC9556748; doi:10.1038/s41386-022-01419-w)
Supplement: Supplementary file 1 — Supplementary Note 1 [file 41386_2022_1419_MOESM1_ESM.docx]

**Notes**

1. We searched the literature on Pubmed using the following terms [(footshock OR quinine) AND self-administration AND (compulsive OR compulsivity)] since January 1^st^ 2020, and retrieved 17 original research articles characterizing animals as “compulsive”.
